# A two-step Bayesian network approach to identify key SNPs associated to multiple phenotypic traits in four purebred laying hen lines

**DOI:** 10.1371/journal.pone.0297533

**Published:** 2024-03-28

**Authors:** Ismalia Bouba, Emiliano A. Videla Rodriguez, V. Anne Smith, Henry van den Brand, T. Bas Rodenburg, Bram Visser

**Affiliations:** 1 Hendrix Genetics Research Technology & Services B.v, Hendrix Genetics, Boxmeer, North Brabant, The Netherlands; 2 Animals in Science and Society, Faculty of Veterinary Medicine, Utrecht University, Utrecht, The Netherlands; 3 School of Biology, University of St Andrews, St Andrews, Scotland, United Kingdom; 4 Adaptation Physiology Group, Department of Animal Sciences, Wageningen University & Research, Wageningen, Gelderland, The Netherlands; University of Life Sciences in Lublin, POLAND

## Abstract

When purebred laying hen chicks hatch, they remain at a rearing farm until approximately 17 weeks of age, after which they are transferred to a laying farm. Chicks or pullets are removed from the flocks during these 17 weeks if they display any rearing abnormality. The aim of this study was to investigate associations between single nucleotide polymorphisms (SNPs) and rearing success of 4 purebred White Leghorns layer lines by implementing a Bayesian network approach. Phenotypic traits and SNPs of four purebred genetic White Leghorn layer lines were available for 23,000 rearing batches obtained between 2010 and 2020. Associations between incubation traits (clutch size, embryo mortality), rearing traits (genetic line, first week mortality, rearing abnormalities, natural death, rearing success, pullet flock age, and season) and SNPs were analyzed, using a two-step Bayesian Network (BN) approach. Furthermore, the SNPs were connected to their corresponding genes, which were further explored in bioinformatics databases. BN analysis revealed a total of 28 SNPs associated with some of the traits: ten SNPs were associated with clutch size, another 10 with rearing abnormalities, a single SNP with natural death, and seven SNPs with first week mortality. Exploration via bioinformatics databases showed that one of the SNPs (*ENAH*) had a protein predicted network composed of 11 other proteins. The major hub of this SNP was *CDC42* protein, which has a role in egg production and reproduction. The results highlight the power of BNs in knowledge discovery and how their application in complex biological systems can help getting a deeper understanding of functionality underlying genetic variation of rearing success in laying hens. Improved welfare and production might result from the identified SNPs. Selecting for these SNPs through breeding could reduce stress and increase livability during rearing.

## Introduction

This study aimed to identify single nucleotide polymorphisms (SNPs) associated with successful rearing to improve welfare and production of laying hens. During rearing, some pullets will die or are culled due to rearing abnormalities. The percentage of pullets that survive until the end of the rearing phase is considered as the rearing success. In white Leghorn layers, rearing success has been shown to be positively affected by clutch size [1]. Furthermore, in wild birds, a swift’s nesting success was directly proportional to clutch size, showing that a larger clutch size during incubation resulted in a higher survival [2]. Another aspect determining rearing success is first week mortality. Besides a performance indicator, first week mortality is also a welfare indicator, as high mortality indicates a potential welfare problem [3]. It has been demonstrated that first week mortality positively correlates with total mortality in layer breeder flocks [4]. First week mortality in broilers has been shown to be affected by season (P<0.001), with the highest mortality (on average 1.18%) being recorded from mid-March until mid-April and the lowest mortality (on average 1.08%) occurring between mid-September and mid-October [5, 6].

Another aspect determining rearing success is culling due to rearing abnormalities. Yeboah et al. [7] gave most attention to day-old chick quality with little attention for rearing abnormalities during the rest of the chick’s life, meaning that rearing abnormalities in relation to rearing success are hardly investigated. A final aspect determining rearing success is natural death. A strong negative correlation has been found between natural death during rearing and rearing success. According to Samkange et al. (2020), natural death in Lohmann Brown layers reared for 12 months from day of placement in the barn onward, contributed to mortality up to 31.6% compared to culls caused by inflammatory conditions (20.9%), trauma (19.3%), cannibalism (16.6%), or retained eggs (11.8%) [8]. A study that was done in a tropical climate showed, mortality of layer breeders (over 78 weeks of age) is less during rearing compared to younger breeders, three times more for the age category 39–58 weeks and 90 times more for the age category 19–38 weeks [9]. Consequently, rearing success of the offspring is expected to be influenced by breeder flock age. Rearing success and its underlying factors are probably affected by genetics. For instance, White Leghorn pullets from three pure lines (Lines 1, 2 and 3) showed differences in survival rate, the survival rates were 47.2, 72.2, and 55.6% respectively [10]. Brinker et al. (2018) examined the heritability of survival rate in three different layer hybrids and found that there are SNPs that can be linked to survival [11]. To better understand the complex biological systems involved in the relationship between SNPs and rearing success, Bayesian networks (BN) can be applied.

BNs are defined as direct acyclic graphs displaying the joint probability distribution of a given set of variables [12–14]. The graphs consist of two elements: i) nodes that represent each one of the variables measured, and ii) links or edges, that represent the statistical relationships or interaction between nodes [12, 14]. The mathematical properties of BNs do not allow loops in the graph structure (e.g., a path over links that starts and ends at the same node) and consequently the graph is acyclic. Relationships within BNs are commonly referred to as a family analogy, where nodes can be parents, represented by those nodes with outgoing links to other nodes, children, represented by those nodes with incoming links from other nodes, and spouses, represented by those nodes that share a common child (or children) [12, 15]. The set of parent and child nodes can be used to put the focus on one variables and to select a small group of variables that have a direct link with the variable of interest [12, 15]. In poultry science, BNs have been implemented to predict the egg production in European quail [16], to further understand the management and welfare of laying hens in different housing systems [17], or to identify factors and processes affecting the quality of composts, specially under tropical weather conditions, using poultry manures [18]. In the field of poultry genetics, only a few studies applied BN algorithms to unravel hidden interactions and informative relationships between a set of genetic or epigenetic features (e.g., differentially expressed genes, differentially methylation regions) [19–22]. For example, the structure of a BN was learnt with some genes previously known to be part of the fatty acid metabolism, allowing the possibility to identify main interactions between them, potentially revealing relevant effects on the metabolic path [19].

The aim of this study was to investigate relationships between SNPs and rearing success of four purebred White Leghorn layer lines by implementing a BN approach. Recorded phenotypic traits determining rearing success were clutch size, first week mortality, rearing abnormalities, natural death, flock age, genetic line, season, and embryo mortality. We applied a Bayesian approach involving four steps: i) reduction of the dimensionality, defined by the SNPs data, by using a Naïve Bayes Classifier algorithm, ii) learning the structure of BNs in order to unravel hidden relationships and interactions between the previously selected set of SNPs and the phenotypic traits, iii) identifying key SNPs associated to each one of the individual phenotypic traits based on direct links with other variables, and iv) usage of different online sources of genetic information to understand the biology behind the findings. The knowledge obtained in this study will allow the further understanding of the key traits related to rearing success together with the underlying genetics behind it, with the possibility of improving animal health and welfare and, therefore, the performance of laying hens in commercial settings.

## Materials and methods

### Phenotypic data

Hendrix Genetics, the Netherlands, provided a dataset of approximately 23,000 rearing batches from four purebred genetic White Leghorn layer lines. Each batch contained eggs of one individual dam. Between 2010 and 2020, eggs per individual dam were collected over a period of 14 days and incubated thereafter. After hatching, the chicks of a batch were identified in the rearing barn with wing bands. The dataset contained different categories of potential explaining factors: animal related factors, environmental factors, genetic factors, and rearing factors. Animal related factors were genetic line, clutch size (the number of eggs laid by a dam within the period of 14 days; as breeder hens are laying eggs continuously, clutch size can be viewed as rather artificial, because it only reflects eggs laid during a specific period), and embryo mortality (the percentage of dead embryos during incubation relative to the number of eggs that was set from a batch at the start of incubation). Environmental factors included season, which was defined as the period a rearing abnormality was registered (categorized into four classes; December, January, February = Winter; March, April, May = Spring; June, July, August = Summer; September, October, November = Autumn). Genetic factors included pedigree and genotype. Rearing factors were flock age (age at which a rearing abnormality was recorded for a pullet in that flock), and the rearing traits first week mortality, rearing abnormalities, natural death, and rearing success. Young birds are referred to as pullets from hatch onward until about 17th weeks of age, after which they are moved to laying barns as adult laying hens.

Rearing success was calculated based on the values of three phenotypic traits and expressed as a percentage according to the following formulae:

RearingSuccess=100–(firstweekmortality+rearingabnormalities+naturaldeath), (1)

where first week mortality is the percentage of pullets that died naturally in the first week, rearing abnormalities is the percentage of pullets that were removed from the rearing barn due to different remarks, and natural death is the percentage of pullets that died naturally after the first week until 17 weeks of age.

#### Genotypic data

Hendrix Genetics provided genotypes of 1,748 dams, which are the maternal parents of the pullets described in this study. The dams were genotyped with the Illumina 60K SNP-chip for chickens and each genotype contained exactly 62,575 SNPs. No genotypes were available on the pullets themselves.

#### Data discretization

The two-step Bayesian approach requires the data to be discretized into a reduced number of categories, considering the distribution of each phenotypic trait. Ideally, data should be discretized into a set of equally distributed categories, where each one of the categories has the same number of observations [23, 24]. This is to avoid potential artefacts of the Bayesian algorithms due to the imbalances between categories. Each one of the genetic lines as well as each one of the seasons were treated as 4 different categories (“Line1” = 0, “Line2” = 1, “Line3” = 2, “Line4” = 3; “autumn” = 0, “spring” = 1, “summer” = 2, “winter” = 3, respectively). Embryo mortality, first week mortality, and natural death had zero as the most frequent value, thus it was not possible to divide the data into equally distributed categories. These 3 variables were encoded as binary variables, where values equal to zero were encoded as zero, while the rest of the values were encoded as one. Clutch size was also discretized into a binary variable based on the distribution of the data points: values lower than or equal to 12 were encoded as zero, while the rest were encoded as one. The rest of the phenotypic traits (rearing success, flock Age, and rearing abnormalities) were discretized into 3-state variables. The quantile discretization method was done, using the “arules” package in R [25].

#### Two-step Bayesian approach: Naïve Bayes and Bayesian networks

In this study, a Naïve Bayes Classifier algorithm was used to reduce the dimensionality of the SNPs data, followed by a BN algorithm, to learn the structure of the networks and to unravel hidden informative interactions between the phenotypic and the SNPs data. Both algorithms are based on the Bayes rule, considering the formula in Eq (2).

P(M|D)=P(D|M)*P(M)/P(D) (2)

where P(M|D) is the posterior probability of the model given the data; P(D|M) is the prior probability of the data given the model; P(M) is the prior probability of the model; and P(D) is the prior probability of the data.

To reduce the dimensionality of the genotypic data and to select relevant SNPs, a Naïve Bayes Classifier algorithm was applied to each of the phenotypic traits of interest: clutch size, first week mortality, natural death, rearing abnormalities, and rearing success, considering each of them as the “y” variable or the class. For each class, the algorithm assumes that the SNPs are independent from one another, and identifies which SNPs are best at predicting the value of the phenotypic trait by providing an ordered list [26]. The 62,575 SNPs were randomly divided into ten groups and the five phenotypic traits were individually used as the classes to select the top 20 most significant SNPs. Dividing the full set of SNPs into ten random sets was done to increase the statistical/computational power while accounting for the whole genetic variability represented by the 62,757 SNPs [27]. The R package “caret” [28] was used to apply the Naïve Bayes Classifier algorithm. Initially the “trainControl” function was set with the “*repeatedcv*” as the method, 10 as the number of folds, and 3 as the repeated k-fold cross-validation number. Subsequently, the “train” function was applied, using “*naive_bayes*” as the method. Finally, the function “varImp” was applied to get the top 20 most important SNPs. These series of steps were repeated for each one of the 10 subsets of SNPs and then used for further analysis in the second step of the approach. If a SNP was found to be among the top 20 most significant ones of more than one phenotypic trait of interest, it was considered as a single entry.

A BN algorithm was used to discover informative relationships and interactions between SNPs and phenotypes, and to identify key SNPs associated with the phenotypic traits by selecting those having a direct link with the variables of interest. BNs allow the possibility of including prior pieces of evidence in the form of a list of links to be blocked. Considering that rearing success was calculated based on natural death, first week mortality, and rearing abnormalities, and to avoid the risk of including confounded features, a list of links to be blocked was included before learning the structure of the BN (Table 1 –Confounded variables). This is because the algorithm might find a link between the confounded features that are already known as a result of previous knowledge, and it will not look for further interactions [29]. Additionally, imbalances between the discrete categories of the data might lead to the discovery of links that should not be present as consequence of an artefact of the BN algorithm. In order to deal with this challenge, a contingency test was applied to every possible pair of phenotypic traits to find possible links to be blocked [24, 30]. The contingency test consisted of a Chi-square test applied to identify the lack of dependency between a given pair of variables. A p-value (P ≥ 0.25) was used as a threshold for the exclusion criteria (Table 1 –Contingency test) as in previous studies [22, 24].

**Table 1:** List of links blocked when learning the structure of the Bayesian network. Considering a link as the interaction between two nodes, the left side of the link represents the parent node (from), while the right side of the link represents the child node (to). The list corresponding to the confounded variables includes links blocked in both directions, from—to and vice versa.

Confounded Variables	Contingency Test
natural death–rearing abnormalities	embryo mortality–first week mortality
rearing abnormalities–rearing success	clutch size–first week mortality
first week mortality–rearing success	embryo mortality–natural death
natural death–rearing success	clutch size–natural death
	season–rearing success

BN structure learning involves the exploration of the search space, using score-based algorithms with the aim of looking for the optimal network that bests fits the data [14, 31]. In the first iteration, the search space starts from an empty or a random graph, followed by the addition, removal, or even reversal of a link between two nodes. The scores of the old and the new network were calculated, compared, and the network with the highest score was kept. In our study, we used a Simulated Annealing algorithm that involves an extra parameter known as temperature, representing the probability of accepting a link that negatively impacts the score metric. As the process iterates, the value of this parameter decreases, reducing the probabilities of accepting the link. The whole process (adding, removing, or reversing links) was repeated until the score was no longer improved or until a stopping criterion was reached (e.g., number of networks visited or a certain period of time) [12, 14]. Banjo (available from http://www.cs.duke.edu/~amink/software/banjo/) was used to learn the structure of the BNs between the phenotypic traits and the SNPs. For each one of the ten datasets (relevant SNPs selected by the Naïve Bayes Classifier plus the nine phenotypic traits), an individual network was built, using a Simulated Annealing algorithm, scoring the networks with the Bayesian Dirichlet score, visiting as many networks as possible in a 30-minute search, and including the list of links to be blocked. Considering that running several times the same search retrieved the same top highest scoring BN, the top graph was used for further analysis. After this step, 10 top highest scoring BNs were learnt, each one of them corresponding to one of the 10 subsets of relevant SNPs plus the phenotypic traits.

**Fig 1:**
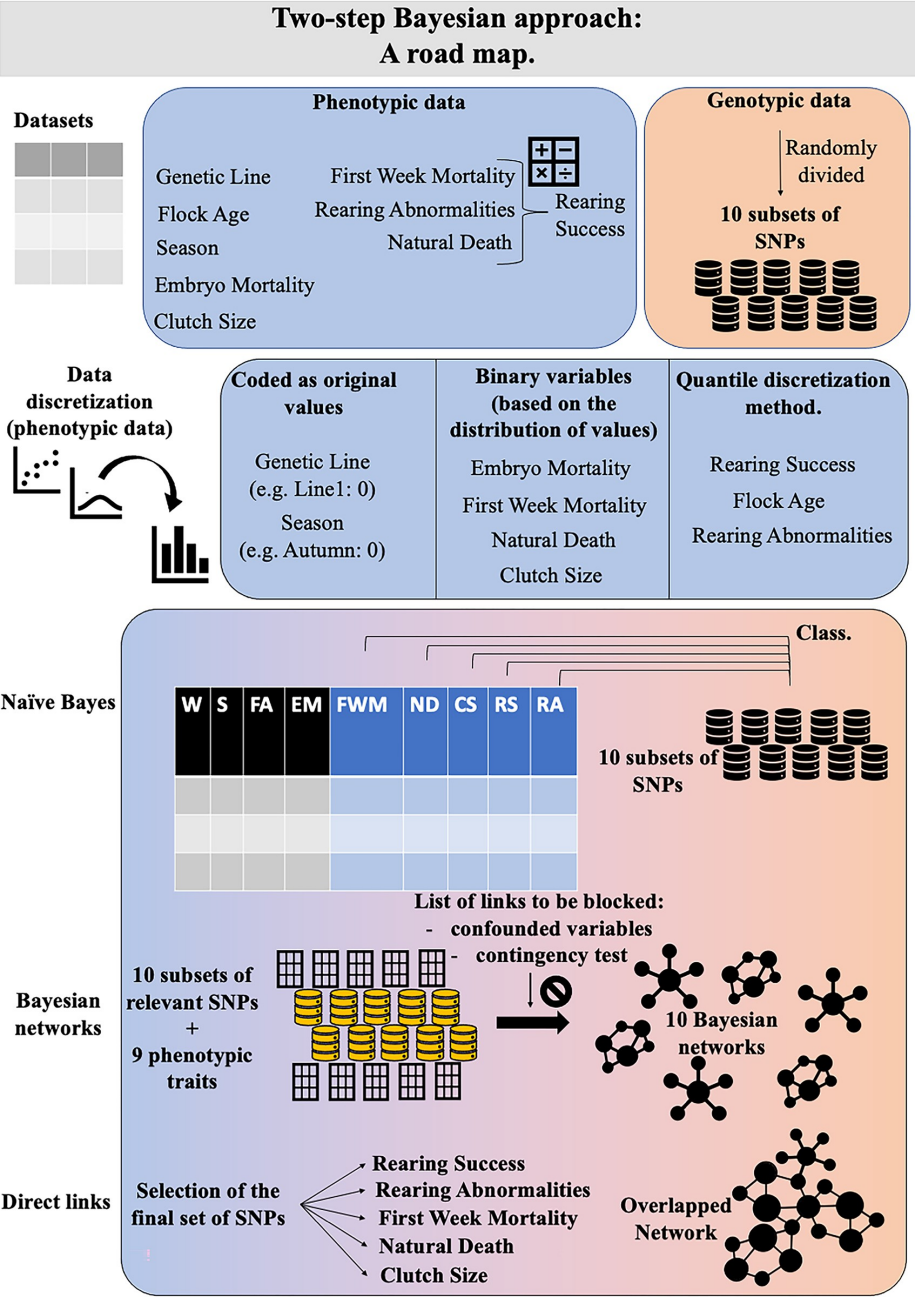
Overall view of the steps taken, and the decisions made, from the data to the overlapped network. For explanation see text. (W = Genetic Line; S = Season; FA = Flock Age, EM = Embryo Mortality; FWM = First Week Mortality; ND = Natural Death; CS = Clutch Size; RS = Rearing Success; RA = Rearing Abnormalities).

Once the structures of the BNs were learnt, those variables having a direct link with the following traits (nodes) of interest were selected: clutch size, first week mortality, rearing abnormalities, natural death, and rearing Success. Both phenotypic traits as well as SNPs closely interacting with the node of interest were selected. A final overlapped network was built by combining the nodes having direct links with the 5 phenotypic traits into one network. Fig 1 displays an overall view of the steps taken and the decisions made to build the overlapped network.

### Biological significance

The chromosomes and positions of the key SNPs were used to search for the ENSEMBL gene IDs of these SNPs in the chicken’s genome of “BEDTools” [32] by using the “closest” function that searches the nearest region in the genome having an Ensemble ID. Furthermore, the ENSEMBL gene IDs were loaded into Database for Annotation, Visualization, and Integrated Discovery (DAVID) bioinformatic tool [33, 34] to obtain their functional annotations. DAVID provides an individual table (the Functional Annotation Table) with the corresponding annotation of the gene, as well as Gene Ontology (GO) terms, Kyoto Encyclopaedia of Genes and Genomes (KEGG) pathways, protein-to-protein interactions, and other pieces of information regarding their functionality. With these pieces of information, DAVID searches for overrepresented terms, using all SNPs being part of the overlapped network (Functional Annotation Clustering), so that these terms can be used as possible tags of the main biological processes associated to the relevant set of SNPs. Considering the SNPs with genetic attributes (e.g., gene symbols), we searched for KEGG pathways associated to stress in our animal model (*Gallus gallus*). The aim was to identify possible links between the relevant SNPs, the phenotypic traits, and stress (without taking into consideration the nature of it). We used the KEGG online bioinformatic resource (kegg.jp) and we searched for pathways using “stress” as the key word and “Gallus gallus” (gga) as the animal model. A total of 17 pathways were retrieved as related to stress, and the annotated SNPs were individually searched in each one of these pathways. Those SNPs that were found to be part of stress pathways were used for further exploration of their biological implications. Finally, the STRING bioinformatic resource (Search Tool for the Retrieval of Interactive Genes, version 11.5 (https://version-11-5.string-db.org/) was used to search for further predicted network interactions between the SNPs related to stress pathways and other proteins. STRING provides networks of predicted protein-to-protein interactions and they can come from different source of evidence, such as databases, experiments, text mining, among others [35]. Finally, these pieces of information were combined into a coherent story to further understand the underlying biological processes, the mechanisms, and the implications related to the SNPs in the context of rearing success traits.

## Results

### Overlapped network: SNPs associated to rearing success traits

**Fig 2:**
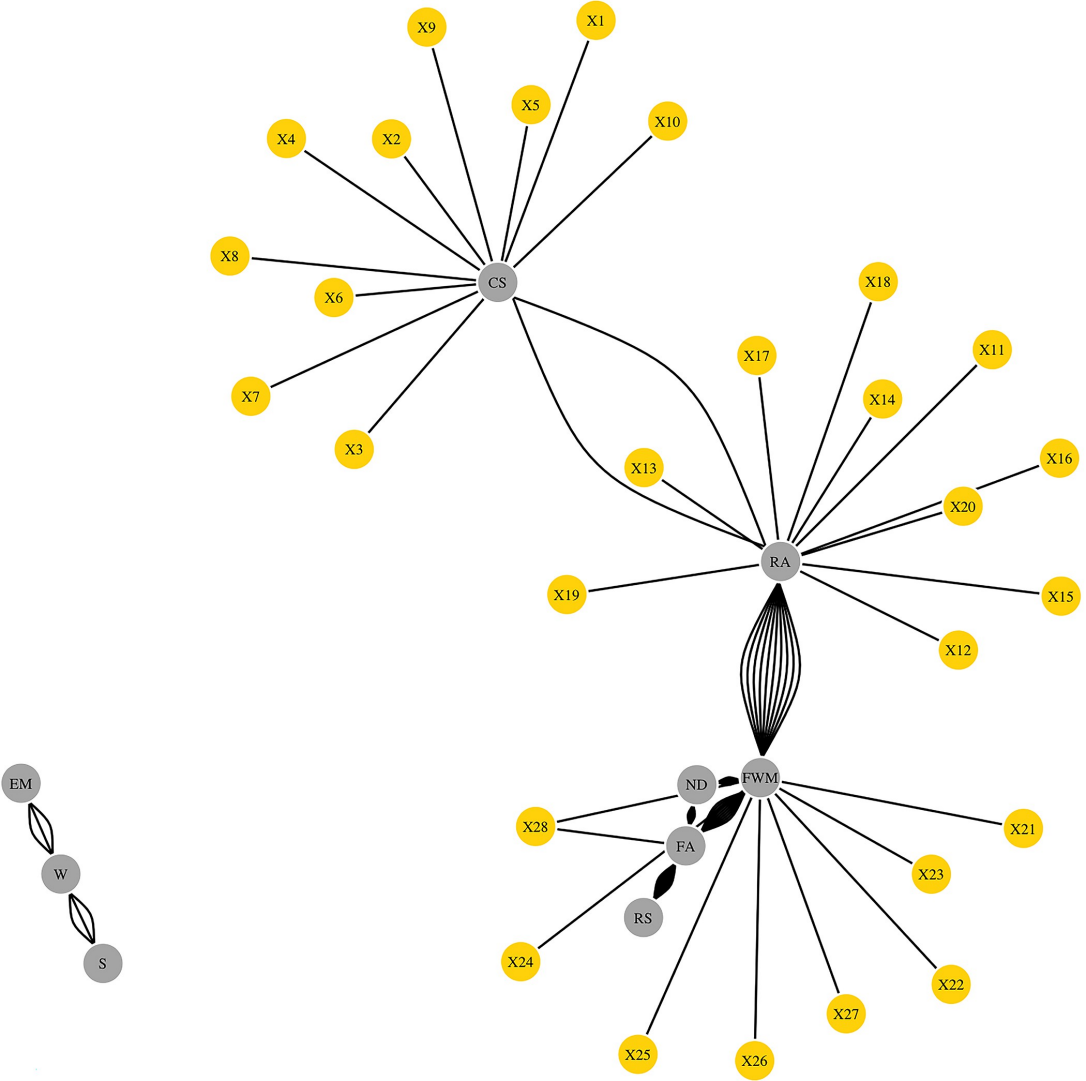
Overlapped network of phenotypic traits (clutch Size, CS; rearing abnormalities, RA; first week mortality, FWM; natural death, ND; Flock Age, FA; rearing Success, RS; Embryo mortality, EM; Genetic Line, W; Season, S) and SNPs in four purebred genetic lines of White Leghorn layers. Phenotypic traits are represented by the grey nodes, while SNPs are represented by the yellow nodes. Connections between the two types of nodes and within the phenotypic traits represent links or edges, which are the probabilistic dependencies in the network.

Fig 2 shows the overall structure of the overlapped network of the rearing traits and the SNPs. All phenotypic traits were included in the overlapped network, but not all of them showed informative and functional links to the SNPs. Based on the structure of the network, two distinct groups of phenotypic traits could be observed: clutch size, rearing abnormalities, first week mortality, natural death, flock age, and rearing success together composed one of the groups, while embryo morality, genetic line, and season composed the other group. This last group of 3 phenotypic traits did not interact with any of the SNPs, however, it is important to bear in mind that only those SNPs interacting with rearing success traits (clutch size, rearing abnormalities, first week mortality, and natural death) were selected. The following interactions were found across the ten sets of top BNs between those traits belonging to the large group containing rearing success: clutch size—rearing abnormalities (CS-RA), rearing abnormalities—first week mortality (RA-FWM), first week mortality—natural death (FWM-ND), first week mortality—flock age (FWM-FA), flock age—natural death (FA-ND), and flock age—rearing success (FA-RS). These traits belonging to the larger group of phenotypic traits were linked to 28 SNPs: clutch size was linked to 10 SNPs, rearing abnormalities was linked to 10 different SNPs, natural death was linked to a single SNP, and first week mortality was linked to seven SNPs (one of which was also linked to flock age). Relationships with more than one link (e.g., RA—FWM) mean that the links was found in multiple BNs.

### DAVID, KEGG, and STRING: Gene ensemble and functional annotations

DAVID Functional Annotation Clustering showed that overrepresented terms across all 16 SNPs with genetic attributes (e.g., gene symbol) were part of two clusters: Cluster 1 was represented by Nucleus (UP KW CELLULAR COMPONENT, p-value = 0.18, Benjamini adjusted p-value = 1), Transcription regulation and Transcription (UP KW BIOLOGICAL PROCESS, p-value = 0.22, Benjamini = 0.68; p-value = 0.25, Benjamini = 0.68, respectively), and Nucleus (GO Term, p-value = 0.39, Benjamini = 1); Cluster 2 was represented by Integral component of membrane (GO Term, p-value = 0.35, Benjamini = 1), Membrane (UP KW CELLULAR COMPONENT, p-value = 0.70, Benjamini = 1), and Transmembrane and Transmembrane helix (UP KD DOMAIN, p-value = 0.75, Benjamini = 1; p-value = 0.89, Benjamini = 1, respectively). Among the SNPs found to be interacting with the traits related to rearing success, three of them were associated with KEGG pathways involved in the stress response, highlighted in the DAVID Functional Annotation Table (Table 2). Such terms particularly relevant to the phenotypic traits as well as stress are regulation of actin cytoskeleton (SNP: *ENAH*); mitophagy–animal (SNP: *MITF*); and *MAPK* signaling pathway, FoxO signaling pathway, and cellular senescence (SNP: *TGFB2*).

**Table 2:** Functional annotation table from the analysis by the Database for Annotation, Visualization, and Integrated Discovery (DAVID) corresponding to only those Single Nucleotide Polymorphisms (SNPs) properly annotated and related to stress pathways. Underlined are highlighted terms particularly relevant to stress and the phenotypic traits.

***ENAH* actin regulator (*ENAH*)**	
KEGG PATHWAYS	Regulation of actin cytoskeleton.
**Melanogenesis associated transcription factor (*MITF*)**	
KEGG PATHWAYS	Mitophagy–animal, Melanogenesis.
**Transforming growth factor beta 2 (*TGFB2*)**	
KEGG PATHWAYS	*MAPK* signalling pathway, Cytokine-cytokine receptor interaction, *FoxO* signalling pathway, Cell cycle, Cellular senescence, *TGF-beta* signalling pathway.

**Fig 3:**
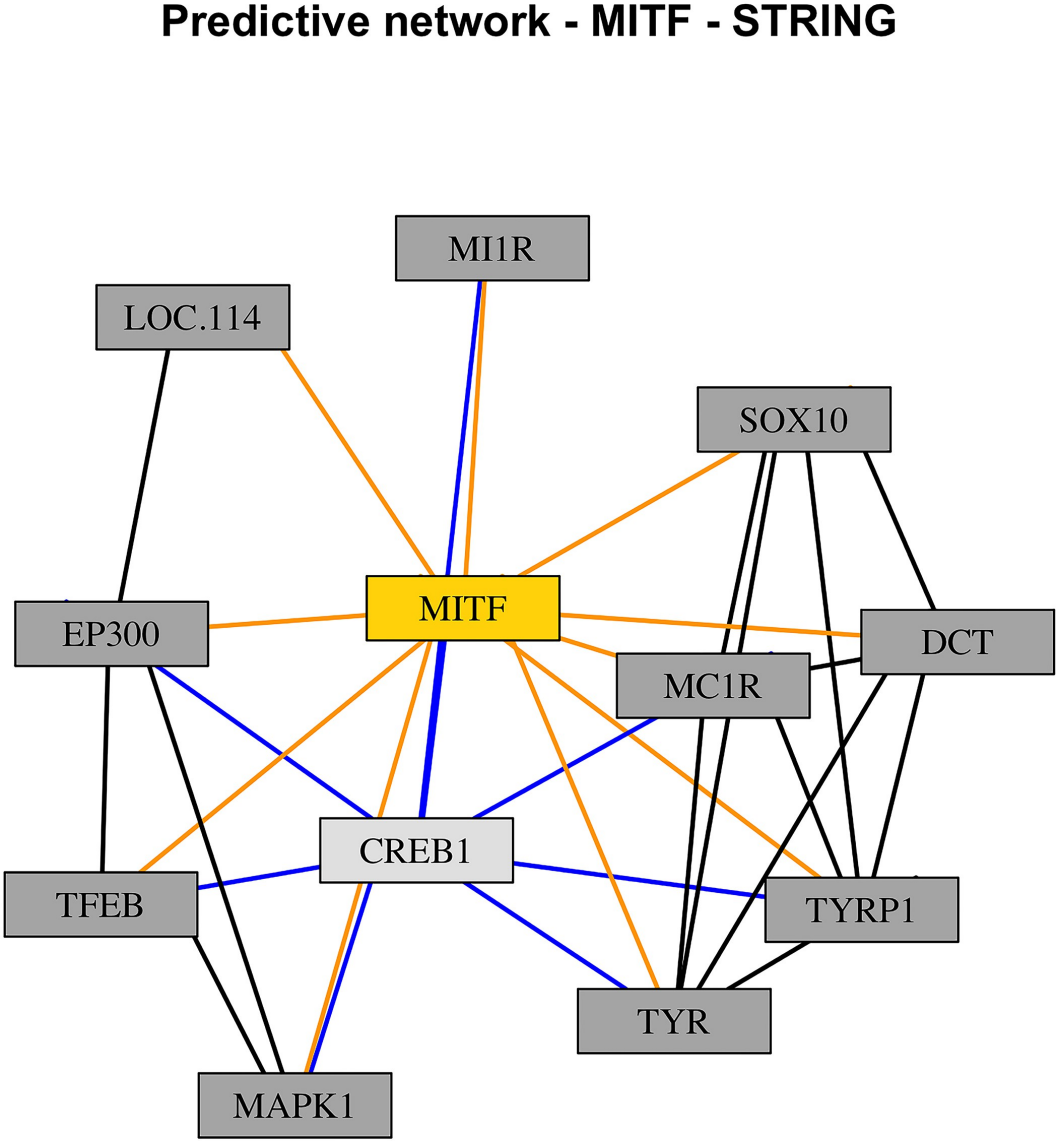
STRING predicted network of *MITF*. Nodes (rectangles) represent proteins, links represent connections identified from previous studies or from curated databases.

**Fig 4:**
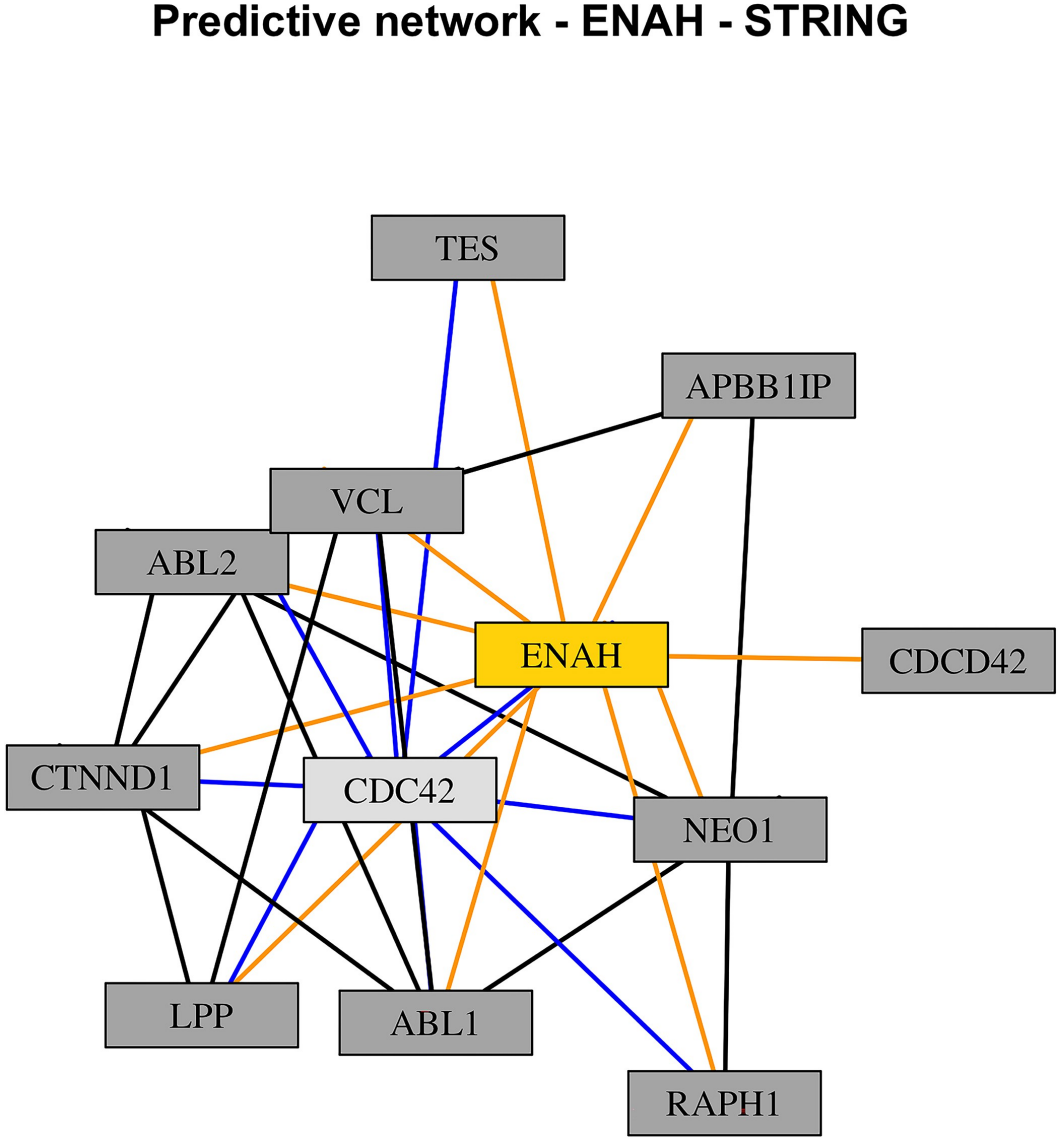
STRING predicted network of *ENAH*. Nodes (rectangles) represent proteins, links represent connections identified from previous studies or from curated databases.

**Fig 5:**
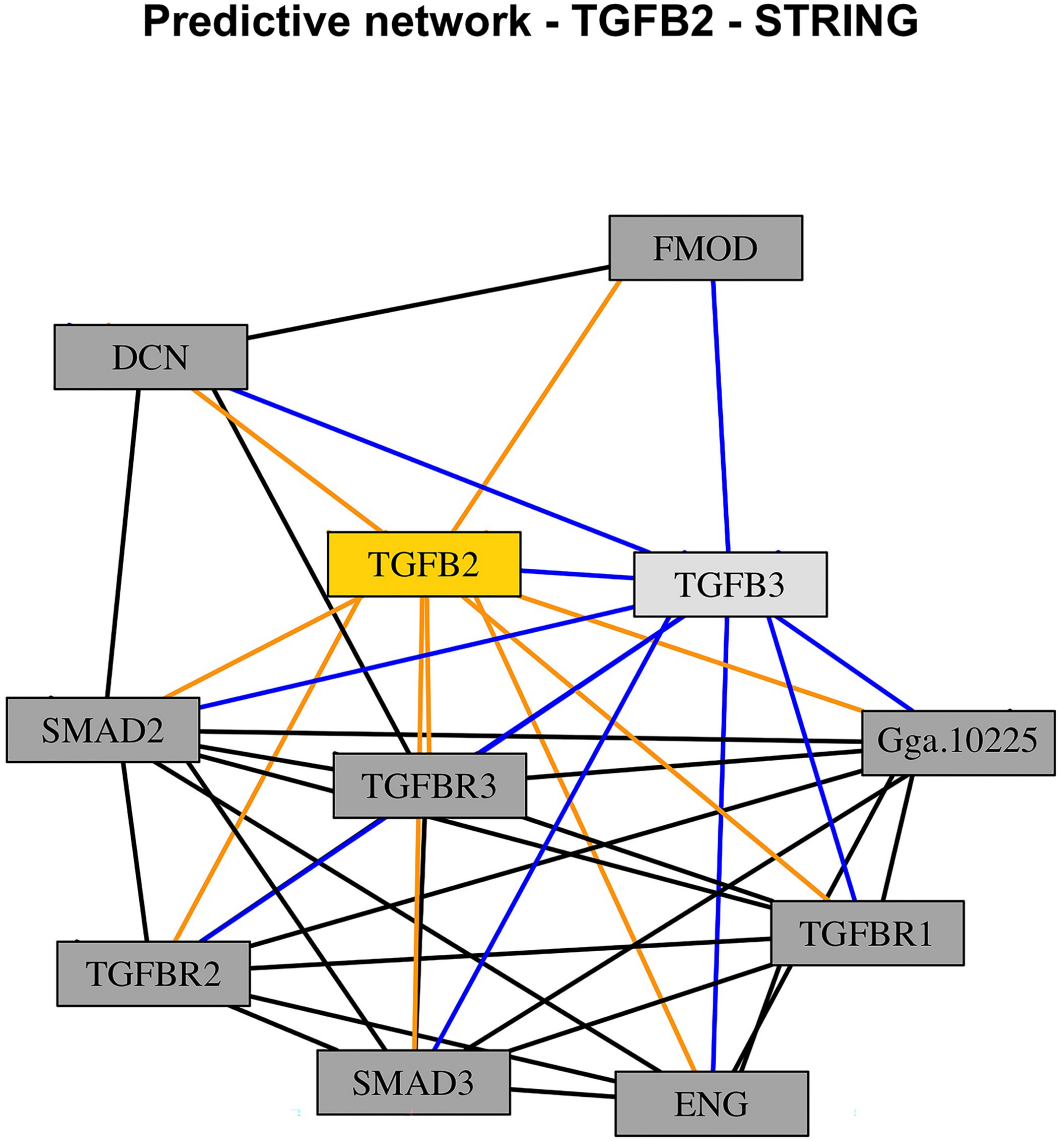
STRING predicted network of *TGFB2*. Nodes (rectangles) represent proteins, links represent connections identified from previous studies or from curated databases.

Since the SNPs *MITF*, *ENAH*, and *TGFB2* were found to be part of at least one of the 17 KEGG pathways (Table 2, KEGG pathways highlighted in bold), further potential connections between them and other proteins were found by using the STRING bioinformatic resource. Three different networks were built, one per each SNP, as shown in Figs 3–5. In each one of the networks, key proteins were identified based on their degree of connectivity (the higher the number of connections, the more important the node is). SNP *MITF* interacted with ten other proteins, and within this network, the protein *CREB1* interacted with seven other proteins (Fig 3, light gey node with blue links); *ENAH* interacted with ten other proteins, and the protein *CDC42* interacted with nine other proteins (Fig 4, light gey node with blue links); finally, *TGFB2* interacted with nine other proteins, and the protein *TGFB3* interacted with ten other proteins (Fig 5, light gey node with blue links). Considering their degree of connectivity, *CREB1*, *CDC42*, and *TGFB3* represent three major hubs of the predicted networks.

Yellow nodes on Figs 3–5 (*MITF*, *ENAH*, and *TGFB2*) represent the SNPs found with the two-step Bayesian approach and their corresponding links with other proteins are highlighted in orange (*MITF* interacted with 10 other proteins; *ENAH* interacted with 10 other proteins; *TGFB2* interacted with 9 other proteins). Considering their degree of connectivity, proteins with the highest number of interactions are highlighted in light grey and their corresponding links are highlighted in blue (Fig 3, *CDC42*, interacted with 9 other proteins; Fig 4, *CREB1*, interacted with 7 other proteins; Fig 5, *TGFB3*, interacted with 10 other proteins). These proteins represent major hubs of the networks provided by STRING.

## Discussion

Rearing success involves features as clutch size, first week mortality, rearing abnormalities, and natural death. The structure of the BN included all phenotypic traits, divided into two distinct clusters. SNPs highlighted as influencing rearing success by BN analysis were mostly associated with clutch size, rearing abnormalities, and first week mortality.

The identification of clutch size as a connected node in the overlapped network on Fig 2, may indicate clutch size as an important trait to consider for improving rearing success. The connections between clutch size and rearing abnormalities acted as the primary bridge between incubation and rearing phase. This connection might be because removing animals from the barn due to rearing abnormalities resulted in a lower rearing success. Additionally, the strong connection between first week mortality and rearing abnormalities might suggest first week mortality is an essential performance indicator [3].

Furthermore, first week mortality interacted with natural death and flock age (strongest association with rearing success), implying that breeder flock age during rearing can influence rearing success of the pullets. A study found that layer breeders older than 78 weeks had a lower risk of natural death compared to younger breeders [9].

After applying the two-step BN approach, a set of 28 SNPs were found to be closely associated with some of the phenotypic traits. DAVID Functional Annotation Clustering did separate the SNPs into two clusters but had generally non-significant associations with functions with both clusters. However, the DAVID Functional Annotation Table revealed corresponding annotations and KEGG pathway for 16 of the SNPs. Among them, three SNPs, *MITF*, *ENAH*, and *TGFB2* had KEGG pathways related to stress in chickens, and they are of interest to the current study (Table 2 –terms highlighted in bold). Although the main focus of our study was particularly on stress, it is important to bear in mind that these SNPs (MITF, ENAH, and TGFB2) are also playing other roles in the physiology of the chickens, as seen in Table 2 (not highlighted terms). Stress is one of the main multifactorial problems the poultry industry is facing nowadays, mostly because of the housing and breeding conditions and potentially worsen by the global warming phenomenon [36–39]. Additionally, there are several other events and/or conditions that might be stressful for poultry species, such as transportation or social interactions [40–42]. Initially, these three SNPs had a direct link with clutch size, which could be considered as the starting point of the life cycle of poultry in commercial settings. Due to this potential connection between rearing success and stress based on those KEGG pathways found by the DAVID tool, thereafter, we used the STRING database to look for proteins that have associations with the SNPs of interest. The reason behind this was to, on the one hand, increase the number of genetic factors involved in the complex biological system, and, on the other hand, to address the potential influence of stress in the lives of laying hens during the rearing phase. Complex networks were found by STRING, and three proteins were found as major hubs of these networks based on their degree of connectivity: *CREB1* interacted with 7 other proteins, including *MITF*, *CDC42* interacted with 9 other proteins, including *ENAH*, and finally, *TGFB3* interacted with 10 other proteins, including *TGFB2*.

Regarding the biology behind these SNPs and proteins, some of them have been involved in embryonic development, neural development, performance parameters, while other have been identified to play a role in reproduction [43–49]. Initially, it is important to highlight that the three SNPs displayed a functional interaction with clutch size, and according to the consensus network, this node had a close direct relationship with rearing abnormalities. Although clutch size is mostly relevant when it comes to reproduction, as it represents the number of eggs laid in a period of 14 days, Bouba et al. (2023) indicated there is a weak positive Pearson’s correlations (R) between (clutch size and rearing success, R: 0.24) and a strong negative R between (rearing abnormalities and rearing success, R: -0.85) which suggests that smaller clutch size may be related with more rearing abnormalities [1]. When it comes to development, *TGFB2* and *TGFB3* belong to the transforming growth factor family, and they are involved in different type of mechanisms and processes [48–50]. They regulate chondrocyte differentiation and hypertrophy as well as the morphogenesis of digits in the limbs, as chicks suffer from dyschondroplasia when the *TGF* levels are low, or the presence of *TGF* in ectopic regions leads to the formation of extra digits in the avian limbs [49, 50]. *ENAH* and *CDC42* have been reported to have a role in the vascular system and the gaseous exchange of the chorioallantois membrane during embryonic development and in defining the destiny of cells during the somatic segmentation, as the absence (or low levels) of *CDC42* leads to the differentiation of cells into epithelial cells, while the presence of *CDC42* benefits the preservation of mesenchymal cells [44, 46]. *MITF* and *CREB1* have also been reported to have some effects on development: *MITF* has been identified in chickens as well as quail with different skin and feather colours, while *CREB1* is involved in neural and retinal development [43, 47]. Considering the role of these genes and proteins in development, reproduction, and performance, changes in their expression levels, pre-processing, and/or functionality might lead to rearing abnormalities and increasing the odds of dying out of a natural death early in life or later during the rearing phase. The overlapped network (Fig 2) showed some serial connections between clutch size, rearing abnormalities, first week mortality, and natural death. As previously described, some of the SNPs and proteins might have impacts on the visible phenotype, while others might also have hidden consequences not visible to the human eye, especially during embryonic development, but still affect rearing abnormalities, first week mortality, and natural death. These consequences might represent a potential link with stress, as they all are welfare-related issues that birds will face during their life. Even though the chickens are not exposed to an external source of stress, such as heat stress or physical restraint, they are under the influence of a chronic influence of an internal source of information, as they will not be able to express normal behaviour, which is one of the five freedoms of animal welfare [51].

Considering that clutch size represents the capability of laying a certain number of eggs in a particular period, one of the proteins found by STRING has a potential role in reproduction. The protein *CDC42*, that interacted with *ENAH*, has been identified in the hypothalamus and the pituitary of red-feather Taiwan chickens with high egg production rates [45]. Particularly, the gene that encodes for this protein was upregulated in contrast with those chickens with low egg production rates [45]. According to Chen et al., there were multiple biological processes associated with this gene, such as cellular physiology, cell communication, growth, differentiation, and adhesion, regulation of cellular and physiological processes, as well as some KEGG pathways, such as focal adhesion, adherent junctions, tight junctions, *MAPK* signalling pathway, among others [45]. In commercial settings, most commercial hens lay almost 14 eggs in a period of 14 days, but some of them do not achieve that goal. Although it seems difficult to increase the number of eggs laid in that particular time frame, it is plausible to think of the possibility of increasing the efficiency of those hens that have lower production eggs. Future studies can be designed to further understand the biological role *ENAH* and its link with *CDC42* in reproduction, with potential emphasis on fertility, follicle development, and embryonic survival.

## Conclusion

It is important to highlight the exploratory nature of our study, and the two-step Bayesian approach can be seen as an alternative to more traditional widely used genetic approaches, such as Genome-Wide Association Studies (GWAS). Both strategies can lead to the discovery of new hallmark genetic features related to phenotypes of interest. Future research studies can be designed with the aim of using the identified SNPs in commercial setting and evaluate whether they can be used to improve animal health and welfare, with indirect implications on reproduction, survival, and the performance of laying hens.
